# Clinical Characteristics and Risk Factors for Chronic Pulmonary Aspergillosis in Patients with Nontuberculous Mycobacterial Pulmonary Disease

**DOI:** 10.3390/jcm15041561

**Published:** 2026-02-16

**Authors:** Ming Wang, Xia Yu, Hairong Huang, Hongfei Duan

**Affiliations:** 1 Department of Tuberculosis, Beijing Chest Hospital, Capital Medical University, Beijing 101149, China; wangming20001102@mail.ccmu.edu.cn; 2 National Clinical Laboratory on Tuberculosis, Beijing Key Laboratory for Drug-Resistant Tuberculosis Research, Beijing Chest Hospital, Beijing Tuberculosis and Thoracic Tumor Institute, Capital Medical University, Beijing 101149, China; yuxiasmart@163.com (X.Y.); huanghairong@tb123.org (H.H.)

**Keywords:** nontuberculous mycobacterial pulmonary disease, pulmonary aspergillosis, coinfection, *Mycobacterium avium* complex, *Mycobacterium abscessus* complex

## Abstract

**Background:** The incidence of patients with nontuberculous mycobacterial pulmonary disease (NTM-PD) complicated by chronic pulmonary aspergillosis (CPA) has been increasing. CPA is known to be associated with complex treatment regimens and a poor prognosis. However, data from mainland China remain scarce. This single-center retrospective study aimed to evaluate the clinical characteristics, risk factors, and prognoses of patients with concurrent CPA and NTM-PD. **Methods:** We conducted a retrospective review of the medical records of 248 patients diagnosed with NTM-PD. Risk factors for CPA were analyzed via multiple logistic regression, followed by survival analysis. **Results:** Among the 248 patients with NTM-PD, 66 (26.6%) were diagnosed with CPA. Independent risk factors for NTM-PD and CPA coinfection included male sex (OR 2.13, 95% CI: 1.03–4.47), dyspnea (OR 27.9, 95% CI: 4.24–570), cavity (OR 5.95, 95% CI: 2.76–13.9), use of oral corticosteroids (OR 4.28, 95% CI: 1.13–16.6), and interstitial lung disease (OR 15.5, 95% CI: 1.89–361). The wide confidence intervals for some risk factors reflect limited precision. The Kaplan–Meier survival curves indicated a significant divergence between the NTM-PD group and the NTM-PD with CPA group (log-rank test, *p* = 0.00039). However, the adjusted hazard ratio was not statistically significant (HR 2.01, 95% CI: 0.66–6.12, *p* = 0.217). **Conclusions:** In patients with NTM-PD, the presence of concurrent CPA was associated with higher unadjusted mortality. Clinicians should maintain a high index of suspicion for CPA to ensure prompt diagnosis and treatment, particularly in high-risk individuals.

## 1. Introduction

Nontuberculous mycobacterial pulmonary disease (NTM-PD) is a chronic respiratory infection whose prevalence has sharply increased worldwide, affecting both high- and low-income settings [1]. In China, the *Mycobacterium avium* complex (MAC) and *Mycobacterium abscessus* complex (MABC) dominate the epidemiological landscape, collectively accounting for approximately 84% of all culture-positive cases (MAC ≈ 61% vs. MABC ≈ 23%) [2]. NTM-PD caused by MABC and MAC is notoriously challenging to treat, with cure rates of only 36.1–56.1% and 68.1%, respectively [3,4,5]. Therefore, identifying risk factors associated with treatment outcomes is essential to enhance the success rates of NTM-PD.

Chronic pulmonary aspergillosis (CPA) is a slowly progressive infection caused by *Aspergillus* species. Previous studies have explored CPA as a complication of NTM-PD [6]. It is also one of the most common complications following the treatment of pulmonary tuberculosis [7]. Recent years have seen an increase in the incidence of NTM-PD accompanied by CPA. CPA is associated with disease progression, elevated mortality rates, and increased therapeutic complexity [8,9,10,11].

However, the majority of published data originate from Japan [12], where MAC accounts for the overwhelming majority of NTM-PD isolates. The conclusions from these studies may not be directly transferable to China, where MABC accounts for a larger proportion of cases. MABC exhibits distinct clinical characteristics and therapeutic responses compared to MAC. We hypothesized that the risk profile and prognosis of CPA in NTM-PD patients in China differ from those reported in Japanese cohorts. Data from other regions, which exhibit different distributions of NTM species, are limited. Thus, we performed a retrospective analysis of NTM-PD patients in China, a setting in which MAC remains common but MABC comprises a substantially larger proportion of cases.

## 2. Materials and Methods

### 2.1. Ethical Approval

This study was approved by the ethics committee of Beijing Chest Hospital, Capital Medical University (YJS-2025-16).

### 2.2. Patients

**Figure 1:**
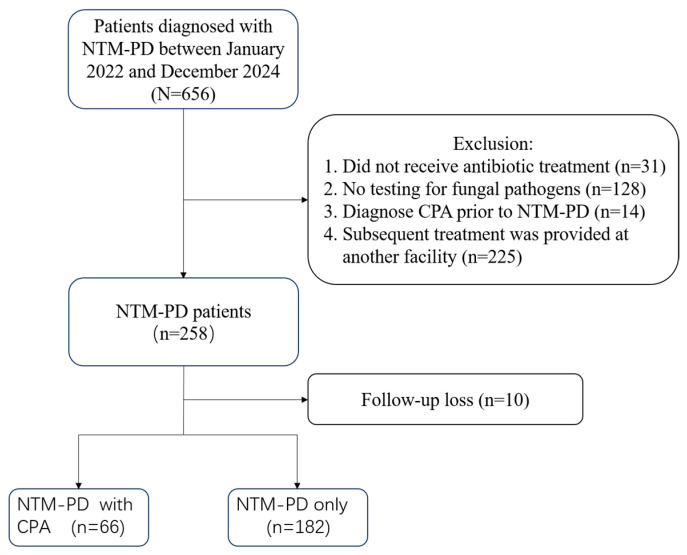
Flowchart of study patients.

This study reviewed the medical records of adult patients (≥18 years) diagnosed with NTM-PD at Beijing Chest Hospital from January 2022 to December 2024. All the patients met the diagnostic criteria for NTM-PD, based on the guidelines by the ATS/ERS/ESCMID/IDSA [13]. We excluded patients who: (1) did not receive antibiotic treatment; (2) underwent no testing for fungal pathogens; (3) were diagnosed with CPA prior to NTM-PD; or (4) received subsequent treatment at another facility (Figure 1).

### 2.3. Study Design

This was a retrospective cohort study. Clinical data including age, sex, smoking history, drinking history, body mass index (BMI), complications, symptoms, use of oral steroid and inhaled corticosteroid, laboratory data, isolated mycobacterial species, radiographic features, and treatment history were collected from the patients’ medical records. Patient follow-up was conducted until 30 September 2025. We defined overall survival as the duration from the diagnosis of NTM-PD until death or censoring. Use of steroids was defined as a daily dose of more than 10 mg or a cumulative dose of more than 700 mg of oral prednisolone or a long-term inhaled steroid [8,10,14,15]. Oral and inhaled corticosteroid use were treated as separate binary variables. Individuals who qualified for both were counted separately in each corresponding variable category.

### 2.4. Diagnosis of Chronic Pulmonary Aspergillosis (CPA)

The diagnostic criteria for Chronic Pulmonary Aspergillosis encompass the following components:

(1) Clinical Symptoms: The presence of respiratory symptoms, including unexplained cough, sputum production, or hemoptysis, persisting for three months or longer.
(2) Radiological Features (Chest imaging revealing one or more of the following): (I) Progressive enlargement of a cavity (II) An intracavitary fungal ball (aspergilloma) (III) Pleural thickening, fibrosis, or infiltrates surrounding the cavity.
(3) Microbiological or Serological Evidence (Laboratory confirmation of Aspergillus infection, demonstrated by at least one of the following):

(I) A positive test for Aspergillus-specific IgG antibodies. (II) Direct microscopy of a respiratory tract specimen showing hyphae consistent with Aspergillus. (III) A positive culture for Aspergillus from respiratory specimens on at least two separate occasions. (IV) Histopathological examination of lung tissue indicating Aspergillus infection [16,17].

A systematic mycological workup was routinely performed for inpatients undergoing evaluation for chronic pulmonary infections. This included direct microscopic examination and fungal culture of respiratory specimens, as well as serological testing for Aspergillus-specific IgG antibodies when available. The Aspergillus IgG assay was accessible for 212 patients (85.5%). Among the 66 CPA patients, the diagnosis was established by positive Aspergillus-specific IgG serology alone in 52 patients (78.8%), one patient was diagnosed based on histopathological examination of lung tissue. 60 patients (90.9%) fulfilled all three diagnostic domains (clinical, radiological, and microbiological), while 6 patients (9.1%) met two domains (clinical and radiological with supportive but non-definitive mycological findings).

### 2.5. Statistical Analysis

All data are presented as median [interquartile range (IQR)] for continuous variables and as number (percentage) for categorical variables. The data were compared using the Mann–Whitney U test for continuous variables, and the Fisher’s exact test for categorical variables. Multiple logistic regression was performed to identify independent risk factors. Variables for inclusion in the multivariate logistic regression model were selected through a two-step process. First, all variables with a *p* value < 0.10 in univariate analysis were considered as candidates. Second, from this candidate pool, variables deemed clinically important based on prior literature and expert recommendation—including age, sex, chronic obstructive pulmonary disease (COPD), interstitial lung disease (ILD), dyspnea, cavity, and oral corticosteroid use—were prioritized for inclusion in the final model. Results are presented as adjusted odds ratios with 95% confidence intervals (CIs). A *p*-value < 0.05 was considered statistically significant. Univariate survival analysis was performed using the Kaplan–Meier method, with groups compared by the log-rank test. The Cox proportional hazards model was also utilized for further analysis. Missing data were present for a minority of variables: BMI (*n* = 5), hemoglobin (HB, *n* = 1), C-reactive protein (CRP, *n* = 1), and erythrocyte sedimentation rate (ESR, *n* = 48). In univariate analyses, participants with missing data for a given variable were excluded from that specific comparison. For the multivariate logistic regression and Cox proportional hazards models, only variables with complete data were included as covariates to maintain a consistent dataset across all models. In this study, the AI language model DeepSeek (version DeepSeek-V3.2) was utilized to assist with writing and debugging portions of the R programming code used for data analysis. All AI-generated code and its analytical outputs were independently validated by a statistician. Statistical analyses were conducted using R (version 4.4.2; The R Foundation for Statistical Computing, Vienna, Austria).

## 3. Results

### 3.1. Patient Characteristics

**Figure 2:**
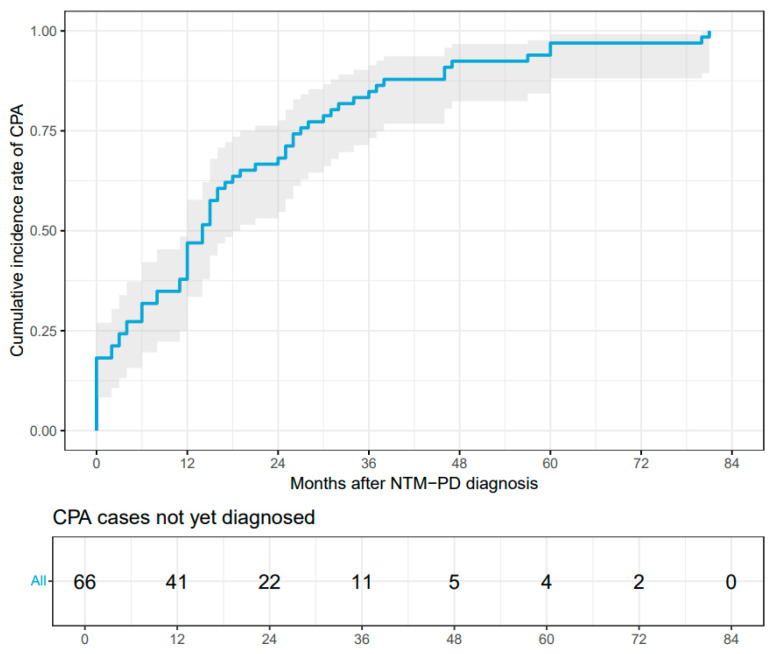
Cumulative incidence of CPA in the NTM-PD with CPA group. The blue line represents the cumulative incidence of CPA over time among all patients in the NTM-CPA group (*n* = 66).

A total of 248 patients with NTM-PD were included in the study (Figure 1). The median observation period was 30 months (interquartile range [IQR]: 20.75–42 months). Among these patients, 66 were diagnosed with CPA during the observation period. This group included 12 cases diagnosed concurrently with NTM-PD and 54 cases diagnosed subsequently. The median time from NTM-PD to CPA diagnosis was 14 months (IQR: 4–26.75) (Figure 2). During the observation period, 59 out of the 66 patients diagnosed with CPA underwent antifungal therapy.

Regarding the distribution of NTM species, MAC accounted for 64.5% of the cases, while MABC represented 27.8%. One patient with CPA was co-infected with both MAC and MABC, and one patient without CPA was co-infected with *M. xenopi* and *M. chelonae*. These two cases were categorized into a mixed infection group. Other identified species included *M. xenopi* (*n* = 7, with 3 developing CPA), M. kansasii (*n* = 8, with 3 developing CPA), *M. gordonae* (*n* = 1, with CPA), and *M. scrofulaceum* (*n* = 1, with 1 case of CPA).

Compared with the non-CPA group, the CPA group showed statistically significant differences across multiple demographic, clinical, and laboratory parameters (*p* < 0.05). The complete comparative data are detailed in Table 1.

### 3.2. Risk Factors for Aspergillus Coinfection

To determine the risk factors for NTM-PD with CPA coinfection, we compared the baseline data of 182 patients diagnosed NTM-PD without CPA and 66 patients with CPA. The univariate logistic regression analysis showed that Age > 60, Male, BMI < 18.5 kg/m^2^, smoking, drinking, COPD, ILD, dyspnea, fever, cavity, diabetes, inhaled corticosteroids, oral corticosteroids, lower albumin and hemoglobin levels and elevated ESR and CRP levels were associated with the condition. In the multivariate analysis, male sex (*p* = 0.041), dyspnea (*p* < 0.001), cavity (*p* < 0.001), oral corticosteroids (*p* = 0.032), ILD (*p* = 0.008) were identified as independent risk factors (Table 2). The odds ratios for dyspnea and ILD were particularly high but accompanied by extremely wide confidence intervals, reflecting the small number of patients with these specific characteristics in our cohort.

### 3.3. Survival Analysis for All-Cause Mortality

**Figure 3:**
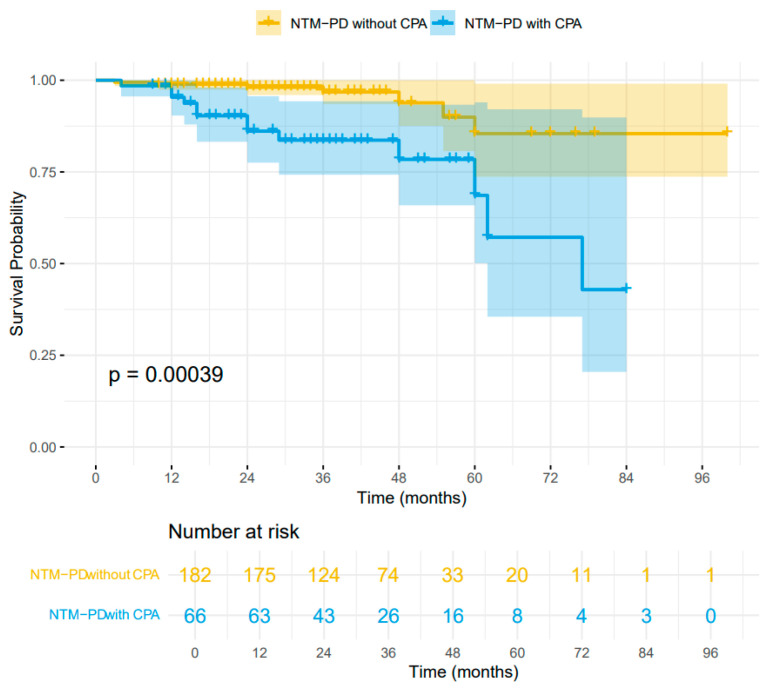
Kaplan–Meier analysis for patients with NTM-PD with or without CPA.

During the follow-up period, 20 patients (8.1%) died. Mortality was significantly higher in the NTM-CPA group compared to the NTM group (19.7% [13/66] vs. 3.8% [7/182]). Kaplan–Meier analysis revealed a statistically significant difference in survival between the two groups (log-rank test, *p* = 0.00039) (Figure 3). Univariate Cox regression analysis demonstrated a significant association between CPA and higher mortality in NTM-PD patients (hazard ratio [HR] 4.63, 95% CI: 1.83–11.71, *p* = 0.001). However, this association was not statistically significant after adjustment for sex, smoking, COPD, dyspnea, cavity, ILD, oral corticosteroids and inhaled corticosteroids (HR 2.01, 95% CI: 0.66–6.12, *p* = 0.217), suggesting that CPA was not an independent predictor of mortality after adjustment (Table S1).

## 4. Discussion

In this single-centered retrospective study, we aimed to assess the clinical characteristics, risk factors and prognosis of NTM-PD coinfected CPA in China. The incidence of CPA was 26.6% (66/248). We identified male sex, ILD, the symptom of dyspnea, cavity on imaging, and oral corticosteroids as independent risk factors for CPA in NTM-PD patients.

In our study, the observed comorbidity rate of CPA was 26.6%, which was substantially higher than that previously reported (2.3–16.0%) [8,9,10,11,12,18,19,20]. We hypothesize that this discrepancy may be attributed to regional variations and inclusion standards. Our study included patients diagnosed with both NTM-PD and CPA simultaneously. As a large tuberculosis specialty hospital, Beijing Chest Hospital typically admits patients with more severe or refractory NTM-PD. Previous studies have reported cavity as a significant factor contributing to CPA. In our cohort, the prevalence of cavity (50%) was notably higher compared to rates documented in prior research (18.6–33.6%) [8,9,10,11,20]. This likely serves as the primary reason for the elevated prevalence of CPA in our cohort. Furthermore, the transfer of patients to local institutions for follow-up treatment could introduce bias, potentially contributing to the higher observed rate of CPA in our study.

Previous studies have identified several risk factors for CPA in patients with NTM-PD, including male sex, the use of systemic corticosteroids, cavity, chronic respiratory failure, and interstitial pneumonia. Corticosteroids suppress the activity and function of immune cells, thereby compromising the host’s ability to resist fungal infections. With prolonged or high-dose administration, the immune system’s capacity to recognize and eliminate fungi is diminished, allowing commensal or environmental fungi to initiate an active infection. In NTM-PD, the presence of cavities not only serves as a marker of disease severity but also creates a favorable niche for Aspergillus colonization and the subsequent formation of aspergillomas [21]. ILD is a well-established independent risk factor for CPA [22]. ILD causes extensive architectural distortion and pulmonary fibrosis, often accompanied by traction bronchiectasis. ILD provides the structural groundwork, while NTM infection may further exacerbate local inflammation and epithelial damage, with both processes acting in concert to promote the development of CPA. Dyspnea in these patients may signify more than a non-specific marker of overall illness severity, it could also indicate poorer baseline pulmonary function, broader disease extent, or greater structural damage. These findings suggest that these patients likely represent a subgroup with more severe NTM-PD, thereby exhibiting increased susceptibility to opportunistic infections such as CPA. Male sex emerged as a risk factor in our cohort, aligning with prior data; this may involve factors such as sex hormones, a higher burden of smoking-related lung disease, and specific ILD subtypes in men. The exact pathophysiology requires further elucidation.

The wide confidence intervals observed for dyspnea and ILD reflect statistical imprecision, likely attributable to the low event counts for these variables. This condition increases the risk of overfitting in the regression model. Therefore, while these factors were statistically significant in our model, the true effect size may be less extreme, and these findings require validation in larger cohorts.

While several studies have identified COPD/emphysema, and MABC pulmonary disease as significant risk factors for CPA in NTM-PD patients, our analysis did not establish them as independent predictors in our cohort [8,10,11]. This discrepancy may involve the broad diagnostic spectrum of emphysema as a heterogeneous entity, suggesting that our cohort may have included patients with relatively milder disease. Different types of underlying lung disease possess distinct predisposing potentials for CPA, and in our cohort, other factors may have outweighed the contribution of COPD and emphysema. Regarding mycobacterial species, there are two main points to consider. Firstly, the previous study only assessed the type of bacteria at the onset of treatment. Given that NTM-PD treatment is lengthy, the prolonged treatment itself may have altered the condition of the lungs and even the predominant mycobacterial species [8]. Secondly, our study had a relatively small sample size. Therefore, larger, meticulously designed studies that control for established strong confounders are necessary to definitively ascertain the independent role of specific NTM species.

In China, coinfection with NTM-PD and CPA has rarely been reported. This can be partly attributed to the fact that the Aspergillus IgG antibody test has only become widely available in many hospitals in recent years. The Aspergillus IgG antibody test demonstrated high sensitivity and specificity for diagnosing CPA [23]. In this study, the Aspergillus IgG antibody test was available for 212 patients (85.5%) out of a total cohort of 248. While this represents a substantial proportion, the incomplete availability could introduce detection bias.

Consistent with prior studies, our findings also indicate that patients with concurrent NTM-PD and CPA experience worse outcomes [8,9,10,11,19]. Therefore, early identification and treatment are particularly important. The therapeutic management of CPA includes antifungal therapy, surgical intervention, and interventional procedures. A course of voriconazole or itraconazole lasting six months or longer is recommended to control infection, delay pulmonary fibrosis, prevent hemoptysis, and improve quality of life [17]. In patients with tuberculosis and CPA, studies have reported that a six-month course of voriconazole is associated with superior efficacy and a lower one-year relapse rate [24].

Our study has several limitations. Firstly, the retrospective, single-center design may introduce potential biases. Secondly, the follow-up period was relatively short. Given that both NTM-PD and CPA are chronic diseases, it is possible that some patients may develop CPA in the future. Thirdly, we excluded some patients with mild symptoms or those who did not undergo fungal testing—individuals who are generally less likely to develop CPA—which may have led to an overestimation of the infection rate. Fourthly, although we defined the survival start time as the NTM-PD diagnosis date for all patients to minimize immortal time bias, a residual bias may persist because patients who developed CPA later had to survive the interim period. This likely led to an underestimation of the mortality hazard associated with CPA. Finally, due to the limited number of cases, we did not investigate the relationships among CPA subtypes, treatment regimens and patient prognosis. Future studies could focus on the aforementioned aspects.

## 5. Conclusions

In conclusion, our study revealed that male sex, dyspnea, cavity, oral corticosteroids and interstitial lung disease were independently associated with CPA coinfection in patients with NTM- PD. Although CPA was associated with a significant increase in mortality, it was not an independent predictor after multivariate adjustment. Clinicians should maintain a high index of suspicion for CPA to ensure early diagnosis and treatment, particularly in high-risk individuals.

## Figures and Tables

**Table 1:** Characteristics of the study patients.

Characteristics	Total (*n* = 248)	NTM-CPA Group (*n* = 66)	NTM Group (*n* = 182)	*p*-Value
Age (years) >60	60 (51.25, 69) 118 (47.6%)	63 (57, 71) 39 (59.1%)	58 (51, 68) 79 (43.4%)	0.0078 0.0317
Sex (male)	103 (41.5%)	44 (66.7%)	59 (32.4%)	<0.001
BMI (kg/m^2^) <18.5 *n* = 243	19.83 (17.11, 21.97) 89 (36.6%)	18.87 (16.22, 22.03) 31 (49.2%)	20.02 (17.58, 21.91) 58 (32.2%)	0.0537 0.0439
Smoking	57 (23.0%)	28 (42.4%)	29 (15.9%)	<0.0001
Drinking	25 (10.1%)	11 (16.7%)	14 (7.7%)	0.0541
Pulmonary comorbidities				
Tuberculosis	76 (30.6%)	24 (36.4%)	52 (28.6%)	0.2758
COPD	45 (18.1%)	18 (27.3%)	27 (14.8%)	0.0389
Asthma	3 (1.2%)	2 (3.0%)	1 (0.5%)	0.1737
Lung tumor	9 (3.6%)	2 (3.0%)	7 (3.8%)	1
Interstitial Lung Disease	7 (2.8%)	6 (9.1%)	1 (0.5%)	0.0016
Pneumoconiosis	7 (2.8%)	2 (3.0%)	5 (2.7%)	1
Extrapulmonary comorbidities				
Diabetes mellitus	22 (8.9%)	10 (15.2%)	12 (6.6%)	0.0446
Cardiovascular disease	43 (17.3%)	15 (22.7%)	28 (15.4%)	0.213
Endocrine system disease	11 (4.4%)	1 (1.5%)	10 (5.5%)	0.191
Digestive system disease	20 (8.1%)	9 (13.6%)	11 (6.0%)	0.076
Rheumatic disease	21 (8.5%)	7 (10.6%)	14 (7.7%)	0.493
Kidney disease	4 (1.6%)	1 (1.5%)	3 (1.6%)	1.000
Nervous system disease	7 (2.8%)	1 (1.5%)	6 (3.3%)	0.681
Other tumors	16 (6.5%)	6 (9.1%)	10 (5.5%)	0.239
Inhaled corticosteroids	6 (2.4%)	4 (6.06%)	2 (1.1%)	0.0447
Oral corticosteroids	13 (5.2%)	7 (10.61%)	6 (3.30%)	0.046
NTM species				0.0042
*M. avium* complex	160 (64.5%)	43 (65.1%)	117 (64.3%)	
*M. abscessus* complex	69 (27.8%)	12 (18.2%)	57 (31.3%)	
Mixed infection	2 (0.8%)	1 (1.5%)	1 (0.5%)	
Others	17 (6.9%)	10 (15.2%)	7 (3.8%)	
Radiological features				
Cavity	124 (50%)	55 (83.3%)	69 (37.9%)	<0.0001
Bronchiectasis	177 (71.4%)	44 (66.7%)	133 (73.1%)	0.3427
Nodule	224 (90.3%)	57 (86.4%)	167 (91.8%)	0.2266
Symptom				
Cough	213 (85.9%)	61 (92.4%)	152 (83.5%)	0.0979
Dyspnea	10 (4.0%)	9 (13.6%)	1 (0.5%)	<0.0001
Hemoptysis	57 (23.0%)	20 (30.3%)	37 (20.3%)	0.1238
Fever	50 (20.2%)	23 (34.8%)	27 (14.8%)	0.0011
Weight loss	51 (20.6%)	18 (27.3%)	33 (18.1%)	0.154
Laboratory Test				
ALB (g/L)	38.5 (34.9, 41.7)	35.45 (32.00, 40.10)	39.35 (36.20, 41.90)	<0.0001
HB (g/L) (*n* = 247)	119 (108, 129)	115 (100, 125)	121 (111, 130)	0.0059
ESR (mm/h) (*n* = 200)	23 (7.25, 52.50)	47 (27, 70)	15 (7, 34)	<0.0001
CRP (mg/L) (*n* = 247)	5.62 (1.12, 28.96)	33.43 (8.71, 62.64)	3.41 (0.88, 12.27)	<0.0001
Follow-up Time (m)	30 (20.25, 42.00)	30 (18, 47)	30 (21, 42)	0.9289
Death	20 (8.1%)	13 (19.7%)	7 (3.8%)	0.0002

Note: Data are presented as median (interquartile range) or number (%). NTM, nontuberculous mycobacteria; CPA, chronic pulmonary aspergillosis; BMI, body mass index; COPD, chronic obstructive pulmonary disease; ALB, Albumin; HB, Hemoglobin; ESR, Erythrocyte Sedimentation Rate; CRP, C-Reactive Protein.

**Table 2:** Univariate and multivariate analyses of risk factors for CPA coinfection.

	Univariate OR (95% CI)	*p*-Value	Multivariate OR (95% CI)	*p*-Value
Age > 60	1.88 (1.07–3.36)	0.03	1.07 (0.53–2.14)	0.9
Male	4.17 (2.32–7.7)	<0.001	2.13 (1.03–4.47)	0.041
BMI < 18.5 kg/m^2^	1.95 (1.08–3.53)	0.038		
Smoking	3.89 (2.08–7.33)	<0.001		
Drinking	2.40 (1.01–5.59)	0.047		
COPD	2.15 (1.08–4.23)	0.03	0.97 (0.39–2.27)	>0.9
Tuberculosis	1.43 (0.78–2.58)	0.2		
Asthma	5.66 (0.53–123)	0.14		
ILD	18.1 (3.01–345)	<0.001	15.5 (1.89–361)	0.008
Pneumoconiosis	1.11 (0.16–5.27)	>0.9		
Lung cancer	0.78 (0.11–3.33)	0.8		
Cough	2.41 (0.97–7.32)	0.060		
Dyspnea	28.6 (5.21–533)	<0.001	27.9 (4.24–570)	<0.001
Hemoptysis	1.70 (0.89–3.20)	0.11		
Fever	3.07 (1.60–5.90)	<0.001		
Weight loss	1.69 (0.86–3.25)	0.12		
Bronchiectasis	0.74 (0.40–1.37)	0.3		
Cavity	8.19 (4.15–17.5)	<0.001	5.95 (2.76–13.9)	<0.001
Nodule	0.57 (0.24–1.42)	0.2		
Diabetes	2.53 (1.02–6.18)	0.046		
Inhaled corticosteroids	5.81 (1.11–42.6)	0.038		
Oral corticosteroids	3.48 (1.11–11.2)	0.032	4.28 (1.13–16.6)	0.032
ALB	0.88 (0.83–0.94)	<0.001		
HB	0.98 (0.96–0.99)	0.004		
ESR	1.03 (1.02–1.04)	<0.001		
CRP	1.02 (1.01–1.03)	<0.001		
NTM species		0.4		
*M. avium* complex	Reference			
*M. abscessus* complex	0.57 (0.27–1.14)			
Mixed	2.72 (0.11–69.8)			
Others	1.17 (0.24–4.40)			

Note: BMI, body mass index; COPD, chronic obstructive pulmonary disease; ILD, Interstitial Lung Dis ease; ALB, Albumin; HB, Hemoglobin; ESR, Erythrocyte Sedimentation Rate; CRP, C-Reactive Protein; NTM, nontuberculous mycobacteria; CPA, chronic pulmonary aspergillosis; OR, odds ratio; CI, confidence interval.

## Data Availability

The data presented in this study are available on request from the corresponding author due to ethical requirements.

## Supplementary Materials

The following supporting information can be downloaded at: https://www.mdpi.com/article/10.3390/jcm15041561/s1, Table S1: Univariate and multivariate analyses of risk factors for All-cause mortality.
