# Supplementary material for: Clinical Characteristics and Risk Factors for Chronic Pulmonary Aspergillosis in Patients with Nontuberculous Mycobacterial Pulmonary Disease
Source: J Clin Med. 2026 Feb 16;15(4):1561. doi: 10.3390/jcm15041561 (PMC12942098; doi:10.3390/jcm15041561)
Supplement: Supplementary file 1 [file jcm-15-01561-s001.zip › jcm-4117794-supplementary.pdf]

	Univariable analysis		Multivariable analysis	
	**HR (95% CI)**	**P-value**	**HR (95% CI)**	**P-value**
Male	6.43 (2.15-19.27)	< 0.001	3.80 (0.93-15.51)	0.063
Smoking	3.86 (1.59-9.37)	0.003	0.87 (0.26-2.87)	0.82
COPD	3.17 (1.25-8.07)	0.016	1.39 (0.40-4.81)	0.603
Dyspnea	5.16 (1.69-15.75)	0.004	2.91 (0.76-11.14)	0.119
Cavity	2.46 (0.88-6.86)	0.085	1.32 (0.42-4.19)	0.639
Oral corticosteroids	1.23 (0.16-9.26)	0.839	0.45 (0.04-4.74)	0.504
Inhaled corticosteroids	18.15 (4.93-66.77)	< 0.001	9.62 (1.61-57.64)	0.013
ILD	7.18 (1.6-32.16)	0.01	2.17 (0.34-13.99)	0.414
CPA	4.63 (1.83-11.71)	0.001	2.01 (0.66-6.12)	0.217

Table S1. Univariate and multivariate analyses of risk factors for All-cause mortality.

COPD, chronic obstructive pulmonary disease; ILD, Interstitial Lung Disease; CPA, chronic pulmonary aspergillosis; HR, hazard ratio; CI, confidence interval.
